# Soil attributes drive nest-site selection by the campo miner *Geositta poeciloptera*

**DOI:** 10.1371/journal.pone.0192185

**Published:** 2018-01-30

**Authors:** Ricardo Camargos de Meireles, João Paulo Gusmão Teixeira, Ricardo Solar, Bruno Nery F. Vasconcelos, Raphael B. A. Fernandes, Leonardo Esteves Lopes

**Affiliations:** 1 Pós-graduação em Biologia Animal, CCB, Universidade Federal de Viçosa - Campus Viçosa, Viçosa, Minas Gerais, Brazil; 2 Departamento de Biologia Geral, ICB, Universidade Federal de Minas Gerais, Belo Horizonte, Minas Gerais, Brazil; 3 Instituto de Ciências Agrárias, ICIAG, Universidade Federal de Uberlândia - Campus Monte Carmelo, Monte Carmelo, Minas Gerais, Brazil; 4 Soil Science Department, Universidade Federal de Viçosa - Campus Viçosa, Viçosa, Minas Gerais, Brazil; 5 Laboratório de Biologia Animal, IBF, Universidade Federal de Viçosa - Campus Florestal, Florestal, Minas Gerais, Brazil; Charles University, CZECH REPUBLIC

## Abstract

Substrate type is a key-factor in nest-site selection and nest architecture of burrowing birds. However, little is known about which factors drive nest-site selection for these species, especially in the tropics. We studied the influence of soil attributes on nest-site selection by the campo miner *Geositta poeciloptera*, an open grassland bird that builds its nests within soil cavities. For all nests found, we measured the depth of the nest cavity and the resistance of the soil to penetration, and identified the soil horizon in which the nest was located. In soil banks with nests, we collected soil samples for granulometric analysis around each nest cavity, while in soil banks without nests we collected these samples at random points. From 43 nests found, 86% were located in the deeper soil horizons (C-horizon), and only 14% in the shallower horizons (B-horizon). Granulometric analysis showed that the C-horizons possessed a high similar granulometric composition, with high silt and low clay contents. These characteristics are associated with a low degree of structural development of the soil, which makes it easier to excavate. Contrarily, soil resistance to penetration does not seem to be an important criterion for nest site selection, although nests in more resistant the soils tend to have shallower nest cavities. Among the soil banks analyzed, 40% of those without cavities possessed a larger proportion of B-horizon relative to the C-horizon, and their texture was more clayey. On the other hand, almost all soil banks containing nest cavities had a larger C-horizon and a silty texture, indicating that soil attributes drive nest-site selection by *G*. *poeciloptera*. Thus, we conclude that the patchy distribution of *G*. *poeciloptera* can attributed to the infrequent natural exposure of the C-horizon in the tropical region, where well developed, deep and permeable soils are more common.

## Introduction

Birds select nest-sites by assessing many biotic and abiotic environmental factors (e.g., [1–5]). Among the abiotic factors, the type of substrate chosen for nest construction is particularly important for cavity-nesting birds [6, 7, 8]. However, the attributes of the substrate that drive nest-site selection are still poorly studied for excavating birds, even though the first observations on the subject were published decades ago [9–11]. Although some authors have addressed this topic in recent years, these studies were mostly conducted in temperate regions (e.g., [6, 7, 12, 13, 14, 15, 16]), and seldom in the tropics (e.g., [17]).

Substrates that are harder to excavate demand greater energy expenditure and time for shaping the nest cavity, which in turn can reduce the reproductive success of a bird [6, 7]. Conversely, very loose soils can result in the collapse of a nest-cavity. Therefore, birds might experience a trade-off between cavity stability and ease of excavation [16], this conflict is called Heneberg compromise [18]. Some woodpeckers, for example, select sites for their nest construction depending on the hardness of the substrate, favoring trees with soft wood or rotting branches and trunks [19, 20]. Additionally, for birds that construct their nests within soil cavities, the granulometric composition of the soil—which is related to the ease of excavation—is an important factor that can even determine the geographic distribution of a species [12].

**Fig 1:**
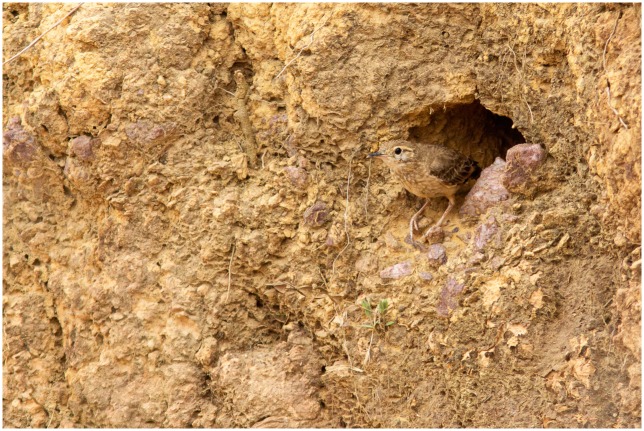
*Geositta poeciloptera* coming out of a monitored nest in the study area—Upper Rio Grande Grasslands, Minas Gerais, Brazil. Photo: Ricardo Mendes.

Among the many tropical bird species that build their nests in soil cavities, the campo miner *Geositta poeciloptera* (Wied, 1830) offers an interesting opportunity for the study of nest-site selection in birds. It is a terrestrial grassland passerine bird that builds its nests within soil cavities either by excavating in steep soil banks, such as gullies, ravines and termite mounds, or by using existing cavities, such as armadillo burrows [21–23] (Fig 1).The species is considered rare, patchily distributed and endemic to the Cerrado biogeographic province [24–26], a savanna-like region, which is one of the world’s biodiversity hotspots [27], where inhabits the “*campo limpo”*, the most threatened formations of the Cerrado [28, 29]. The species is currently undergoing a rapid population decline due to habitat loss and global climate changes [26, 30], and is currently considered globally Vulnerable [31] and Endangered in Brazil [32]. The Brazilian Red List construction follows the same method created by the International Union for Conservation of Nature (IUCN), such as restricted and severely fragmented distribution, also experiencing continuing decline in area of occupancy and quality of habitat [32].

We studied nest-site selection by *G*. *poeciloptera*, investigating which soil attributes can determine nest architecture and the choice of the most suitable place for nesting. We aimed to answer the following three questions: 1) Is cavity depth influenced by soil resistance to penetration? 2) Are soil resistance to penetration and/or soil horizon type driving nest location in steep soil banks? and 3) Which soil attributes (proportion of horizons B and C and soil resistance to penetration) better predict the presence or absence of nests in a given steep soil bank? For the first question, we hypothesize that more resistant soils will have shallower nests. For the second and third questions, we expect that soil horizons with attributes that facilitate nest excavation will be preferentially chosen. This will also influence the choice of steep soil banks used for the construction of nests. Taken together, this knowledge can help us to understand why this species is so rare and patchily distributed, as well as support future efforts for its conservation and management.

## Materials and methods

### Study area

**Fig 2:**
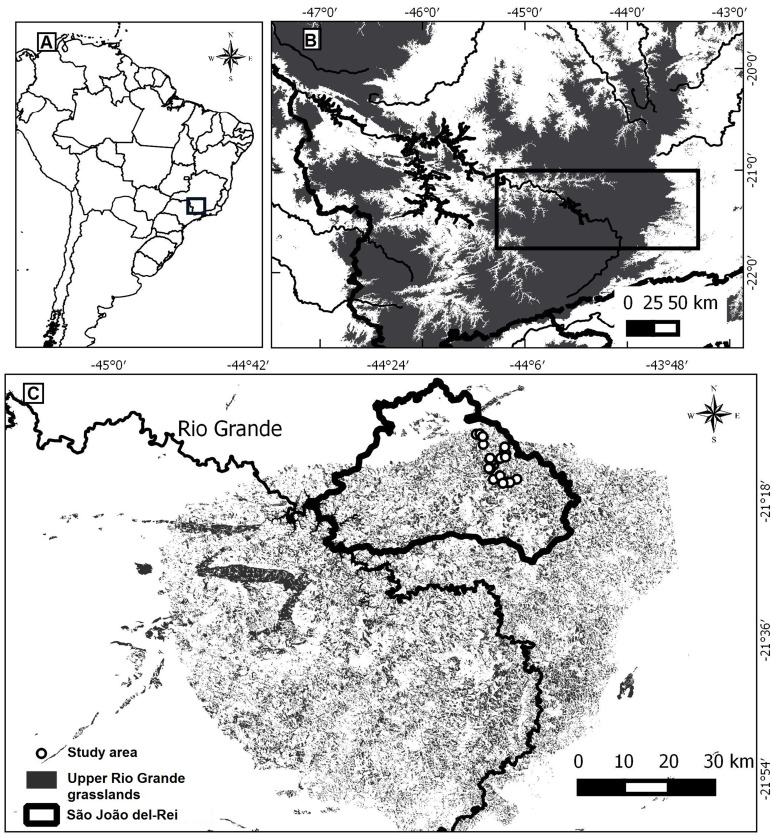
(A) Location of the study area. (B) Detail of the study area, depicting the Rio Grande. Grey areas are above 900 m a.s.l. (C) Detail of the study area, depicting the limits of the municipality of São João del-Rei and the remaining patches of natural grasslands.

The study was conducted in the municipality of São João del-Rei, which is located in the northern portion of Upper Rio Grande Grasslands (URGG), in the southern part of the state of Minas Gerais, Brazil (~21°29’–21°32’S; 43°50’–44°55’W) (Fig 2). The URGG is a large (~12.000 km^2^) mountainous region, with elevation ranging from 900 to 1600 m a.s.l. [33]. The landscape presents a relief that varies from strongly rolling to mountainous, with slopes of inclination between 20 and 75% [34]. The regional geology is characterized by a sequence of metasedimentary rocks of the São João del-Rei Group, of Mesoproterozoic age, with the Prados Formation as the predominant lithostratigraphic unit, constituted predominantly by phyllites, siltstones, and calc-schists rocks [35] These rocks present juxtaposed layers that cause differential resistance to weathering. This differential resistance, associated with intense erosion processes, especially in furrows, results in a landscape with frequent ravine slopes where shallow soils predominate. These ravine slopes are easily identified and distinguished from the pattern of slopes found in adjacent regions, where more developed and deeper soils predominate. These deeper soils have more pronounced permeability, thus facilitating the infiltration of rain water to the detriment of the superficial run off that predominates in the URGG and sculpts the slopes where shallow soils occur (per. obs. by BNFV).

The predominant soils in the study area are Regosols, according WRB/FAO system [36], which are characterized by a sequence of horizons A and C or Cr. Horizons BC and B may also be present when they are less than 10 cm [34]. These soils exhibit a considerable presence of alterable primary minerals and a granulometry with a predominance of coarse fractions such as silt and sand [34]. Other more developed soils are less common, such as Haplic Cambisols or Oxisols (per. obs. by BNFV).

A brief description of the distinct soil horizons identified is presented here for a reader that is not asoil specialist. The A-horizon is the most superficial, and has a greater amount of organic material. The B-horizon is in the subsurface and usually has higher clay content, as well as better structural development (formation of aggregates). The C-horizon, in turn, is identified by its morphological heterogeneity, by the presence of aspects directly related to the soil source material, and is characterized by the predominance of unconsolidated mineral material. For these aspects, the C-horizon differs significantly from the more pedogenized horizons (A and B). The BC-horizon is transitional between B and C, and thus possesses characteristics of both. In the same way, the Cr-horizon possesses characteristics of the C-horizon and structural evidence of the rock of its origin [37].

The predominant vegetation above 800 m comprises natural open grasslands [33]. Although the URGG is located within the Atlantic Forest domain [38], the region would be more properly considered an ecotone, as it is in the transition with the Cerrado, with which the URGG possesses great similarity in flora and fauna [39, 40]. Along the valleys, where soils are deeper and more humid, seasonal semideciduous forests predominate [39].

The local climate is Cwb according to the Köppen classification system, which means it has a humid temperate climate with dry winters and mild summers. The average temperature is 14.3°C during the winter and 17°C during the summer [41]. The dry season is from May to August and the wet season is from September to April, with mean annual rainfall of ~1,500 mm [41].

### Data collection

Data were collected in 2015 and 2016 during the breeding season of *G*. *poeciloptera* in the study area (August to December) [23]. We used three methods for locating nests: 1) active searches in the habitat, inspecting all kinds of structures that could support nest cavities (e.g., ravines, gullies, termite mounds); 2) behavioral observations (e.g., following adults until observing them excavating nest cavities or carrying food for nestlings); and 3) active searches along roads, since the species often nests in road side steep soil banks [23]. For all nests found, we measured the depth of the cavity with a measuring tape and georeferenced their location with a GPS device for monitoring. All procedures performed in this study involving animals were in accordance with the ethical and legal standards and with all necessary permits provided by CEMAVE/ICMBio (50487–1) and CEUA-UFV (53/2015).

### Soil sampling and physical analysis

**Fig 3:**
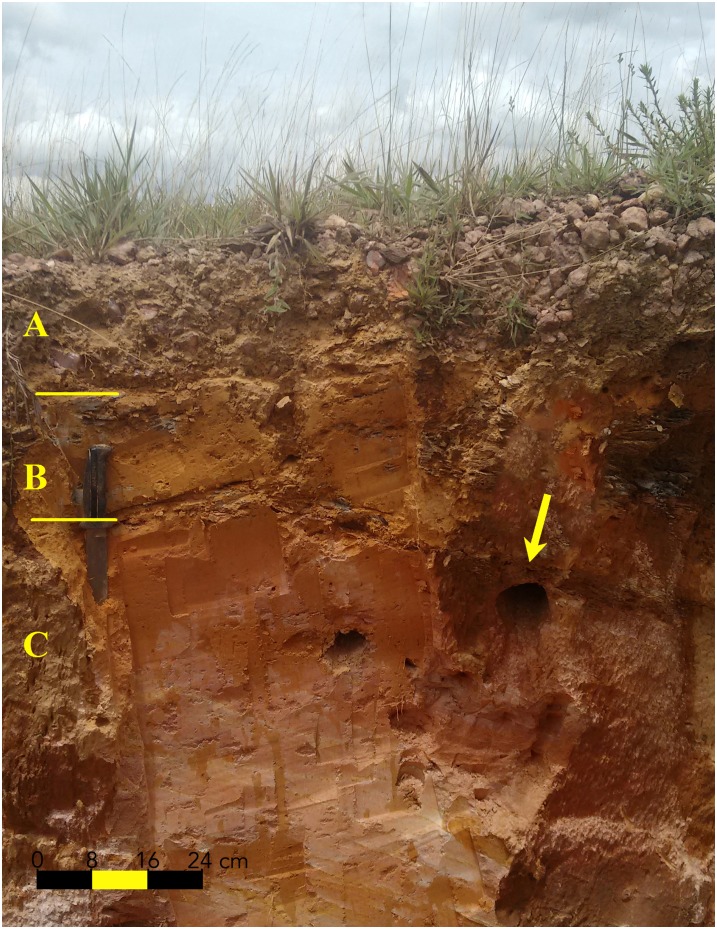
Soil profile showing the identification of the horizons (A, B, and C) containing *Geositta poeciloptera* nest cavities in the Upper Rio Grande Grasslands, southeastern Brazil. The yellow arrow indicates the location of the nest cavity on the horizon C. Photo: RCM.

The soil of each dirt bank was scraped and cleaned of debris and the soil horizons (A, B, BC, C, or Cr) identified according to [42], from the morphological examination of the profiles (Fig 3) and measurements of the depth of each horizon.

After identifying the soil horizons, we collected a soil sample (~150 g) 15 cm from each nest. The particle size distribution was analyzed for the samples following the Ruiz’s [43] protocol. This granulometric analysis aimed to assess the proportion of each soil particle size-class based on sieving and sedimentation techniques, classifying these particles into three groups: sand (2,000–50 μm), silt (50–2μm), and clay (<2 μm) [44]. Results of the analysis of each sample were then plotted in the textural triangles [42], which are then used for the classification of soil texture. We also measured soil resistance to penetration, expressed as the cone index (MPa), which describes the mechanical resistance of the soil to the penetration of a metal rod, and is related to the degree of soil compaction. We measured soil resistance to penetration with a field penetrometer (Wykeham Farrance^™^), by performing four measurements, adapted from Heneberg [16], around each nest (5 cm above, below and on both sides). We also measured the same soil features at random points in steep soil banks that did not possess nest cavities in order to analyze which soil characteristics could be driving nesting sites selection.

In order to avoid interfering in the reproduction of this species, we performed all measurements, sample collections, and soil profile evaluations after nests became inactive.

### Relationship between soil and nests

To evaluate the influence of soil attributes on nest site selection by the campo miner, we randomly drew 25 steep soil banks that harbored nest cavities and 25 steep soil banks that harbored no nest cavity, but that were similar to those possessing nests. To standardize the sample, all banks (i.e. harboring a nest or not) were located in “*campo limpo*”, in places with sparse vegetation, and where the occurrence of the *G*. *poeciloptera* had been recorded (i.e., we checked for the presence of the species in the surroundings of all steep soil banks by playing back the species’ vocalization in each area). Additionally, the non-harboring banks had minimum heights similar to those harboring nests (≥ 51 cm). Once selected, we measured the length of each bank and randomly drew a height and length where a soil sample was taken for subsequent analysis as described above.

### Statistical analysis

We used a simple linear regression with Normal distribution to evaluate the relationship between soil resistance to penetration as the explanatory variable and nest cavity depth as the response variable [45]. Then we performed two logistic regressions, using GLM and binomial error distributions [45] to verify. In the first (local scale), we aimed to understand, using only steep soil banks possessing nests, whether soil resistance to penetration, the ratio of the thickness of horizons (ratio C-horizon / B-horizon), the total height of each bank and the interaction between all variables can explain the presence/absence of nests at a particular point of a given dirt bank. In the second (regional scale), using banks with and without nests, we aimed to understand if a given dirt bank was selected for the excavation of nest cavities based on the proportion of horizons, total height of each bank and resistance to penetration compared to these attributes of steep soil banks without nests. The level of significance adopted in all analyses was 5%. For all models, we tested for attendance of all assumptions such as error distribution suitability (i.e. Normal for the first and Binomial for the two latter), presence of outliers and linear model fit using residual analyses [46]. All analyses were performed in R [47].

## Results

**Fig 4:**
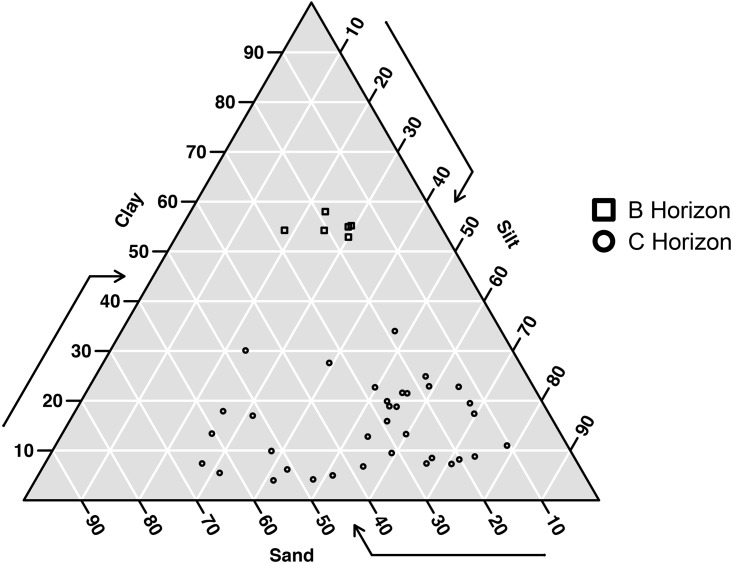
Textural distribution of the soil samples for the 43 nest cavities of *Geositta poeciloptera* found in the Upper Rio Grande Grasslands, southeastern Brazil. Because they have similar textural characteristics, the horizons BC, C and Cr were identified only as C-horizon.

We found 45 active nests (25 nests in 2015 and 20 nests in 2016), but only 43 nests were used in this study since two of them were built within cavities supposedly excavated by the campo flicker *Colaptes campestris* (Picidae). Most of the nests were found in the C-horizon (72%, n = 31), followed by the B-horizon (14%, n = 6), the BC-horizon (11.6%, n = 5), and the Cr-horizon (3%, n = 1). The granulometric analyses showed that horizons BC and Cr, which are transitional horizons, had great similarities with the C-horizon, all possessing high silt and low clay contents. In contrast, the B-horizon had high clay and low silt contents (Fig 4).

**Fig 5:**
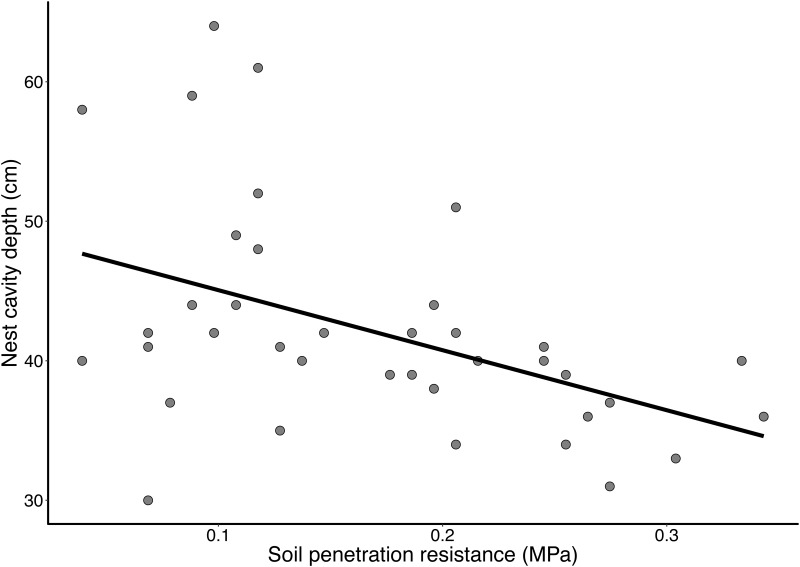
Relationship between soil resistance to penetration and depth of the nest cavity of *Geositta poeciloptera* in the Upper Rio Grande Grasslands, southeastern Brazil (F_1.43_ = 11.42, *P*<0.01, R^2^ = 0.21).

Nest cavity depth was negatively related to soil resistance to penetration (F_1,43_ = 11.42, *P*<0.01, R^2^ = 0.21). As depicted in Fig 5, soils that were more resistant to excavation tended to have shallower nests.

### Influence of horizon thickness and soil resistance on nest-site selection

**Fig 6:**
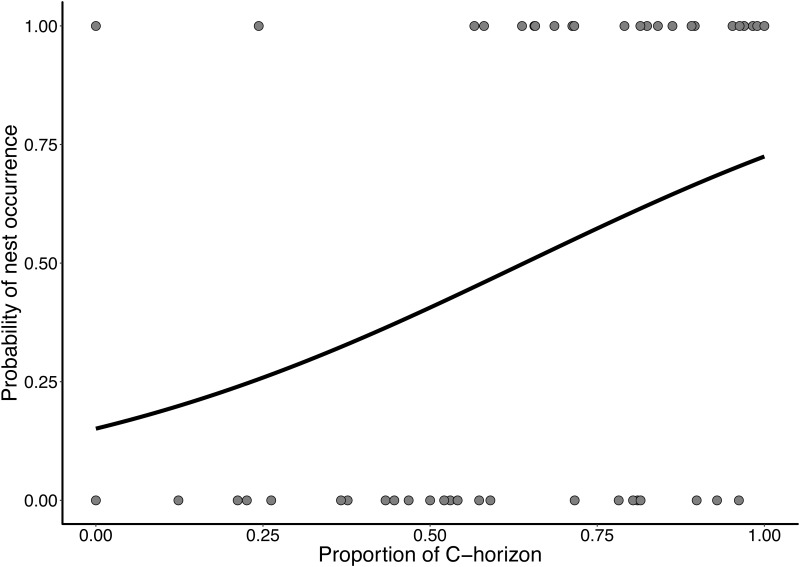
Probability of nest occurrence of *Geositta poeciloptera* according to the proportion of the C-horizon in the Upper Rio Grande Grasslands, southeastern Brazil. 0: steep soil banks without nests; 1: steep soil banks with nests (χ^2^ = 6.11, Df = 48, *P* = 0.013).

**Fig 7:**
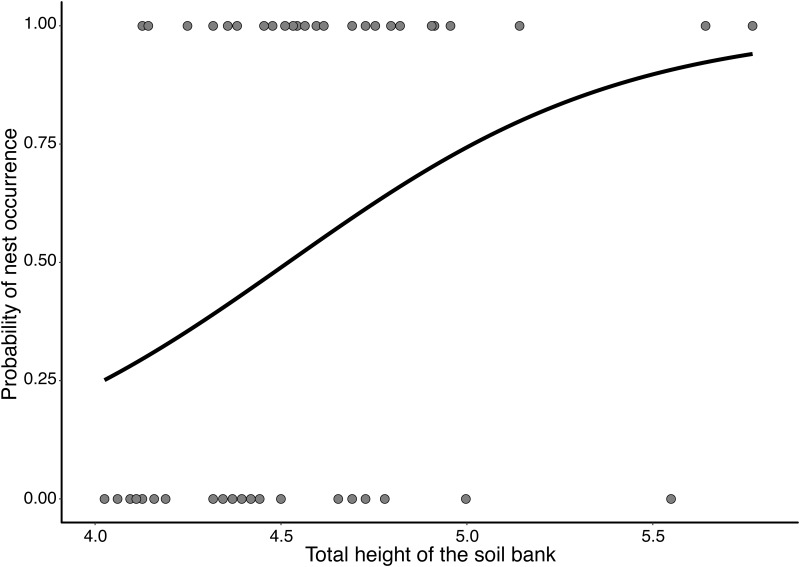
Probability of nest occurrence of *Geositta poeciloptera* according to the total height of the dirt bank in Upper Rio Grande Grasslands, southeastern Brazil. 0: steep soil banks without nests; 1: steep soil banks with nests (χ^2^ = 6.22, Df = 47, *P* = 0.012).

The proportion of C-horizon in the steep soil banks had a positive and significant relationship with the probability of nest presence (χ^2^ = 6.11, Degrees of freedom (Df) = 48, *P* = 0.013; Fig 6), as well as the total height of the bank (χ^2^ = 6.22, Df = 47, *P* = 0.012; Fig 7). However, we did not observe a significant relationship between the presence of nests and soil resistance to penetration (χ^2^ = 1.74, Df = 67, *P*> 0.05). We also did not observe a significant relationship between soil resistance to penetration and the proportion of C-horizon (χ^2^ = 0.035, Df = 68, *P*> 0.05). In short, higher steep soil banks, possessing also a higher C-horizon proportion were more likely to have nests, however, this was independent of the resistance of these soils. Additionally, 40% of the steep soil banks without nest cavities presented a B-horizon that was thicker than the C-horizon (n = 10) and, consequently, soils with a more clayey texture. In contrast, almost 90% of the steep soil banks with cavities had C-horizons larger than B-horizons (n ​ = 22) and, consequently, soils with more silt texture.

## Discussion

Soil resistance to penetration significantly affected nest cavity architecture of *G*. *poeciloptera*, which builds shallower nests at sites with higher soil resistance to penetration. However, this soil attribute does not seem to interfere in the selection of nesting sites, since soil resistance to penetration was found to be similar between steep soil banks with and without nest cavities. Contrarily, soil resistance to penetration is a major factor in nest-site selection for the passerine bank swallow *Riparia riparia* and non passerines blue-tailed bee-eater *Merops philippinus*, the european bee-eater *M*. *apiaster* and the eurasian kingfisher *Alcedo atthis*, all of which select sites with greater or lesser soil resistance, depending on the species requirements and the conflicting requirements of tunnel stability and ease to excavation [13, 16].

Soil granulometry showed to be an important factor for the selection of nesting sites, agreeing with results found for *Alcedo atthis* [7] and *Riparia riparia* [6]. These results are in line with previous studies conducted with swallows, which demonstrated their preference for sites with less clay content, which makes excavation easier [10, 11]. According to [17], soil texture is a parameter closely linked to the selection of nesting sites by the malachite kingfisher *Corythornis cristata*, which selects sites with a higher concentration of silt. In addition, the higher proportion of C-horizon exposed in the soil allows the construction of nests in a higher position—preferential for nesting—which could, for example, reduce the risk of predator accessing the nest, as shown by some studies, ensuring grater breeding success [1, 48, 49]. Other studies with a bee-eater and a swallow pointed to a preference for sandy or sandy-clay soils [12–15], since these soils possess lower humidity and higher aeration, and thus provide adequate levels of O_2_ and CO_2_ within the nest cavity [13, 14]. These observations, and the results of our study confirm that nesting site choice by birds is complex and may involve several factors besides ease of excavation.

Our study, to the best of our knowledge, is the first to evaluate the influence of soil attributes on nesting site selection for any species of Scleruridae, or even any suboscine passerine. A brief visual description of the soil found in a dirt bank with nests of the coastal miner *Geositta peruviana* suggests that the species favors sandy and loose soils [50], but given that this study was based on a small sample size and that it employed no method of soil analysis, nest preferences for *G*. *peruviana* remain disputable.

We demonstrated here that the preferential allocation of nests of *G*. *poeciloptera* to the C-horizon can be attributed, at least in part, to their greater ease of excavation, which is caused by three characteristics of this horizon: 1) low clay and high silt contents; 2) soil consistency, which is soft when dry and loose when moist; and 3) absence of soil structure. Structured soils form aggregates, which are larger units formed from mineral and/or organic particles of different sizes, which gives them greater stability and coherence [51]. In soils with low clay content, the development of structure is impaired, because this is the fraction of greater reactivity in the soil, providing union between distinct particles through chemical interactions [52]. High levels of silt do not contribute to soil structure, because silt particles are not endowed with electric charges and possess low specific surface, making them much less reactive than clay particles. These limited physical and chemical interactions between the solid particles of the soil result in an incipient performance of the cohesive forces between them and, consequently, the soil easily disintegrates when handled.

During the last few decades, the inevitable road cuts necessary for the implementation of a road network in the URGG have dramatically increased the availability of steep soil banks with the C-horizon exposed in the region. Given that nests were found almost exclusively along roads in this study, the possibility that road cuts might have positively impacted the population size of the species in the study area cannot be dismissed, as shown in [8], which in their study demonstrated that the construction of nests by *Cyanoliseus patagonus* is influenced by human interference such as roads and human settlements. Therefore, the execution of controlled cuts in the landscape and the consequent exposure of the C-horizon may be a possible management strategy for the species, although with caution since other birds (e.g., *Alopochelidon fucata* and *Stelgidopteryx ruficollis*) use the same steep soil banks for nesting (pers. obs. by RCM). Besides, this exposure should be carefully planned, since this horizon is very susceptible to erosive processes.

## Conclusion

Soils of the study area have low permeability and show narrow superficial horizons. These soils are subjected to naturally occurring erosive process that produces a landscape full of deep ravines and gullies where the subsurface C-horizon is frequently exposed. Once exposed, the C-horizon becomes the preferred site for nesting by *G*. *poeciloptera* due to the absence of structure and the low performance of cohesive forces between solid particles. This finding is important for understanding the distribution and abundance of *G*. *poeciloptera*, which, although globally rare, is surprisingly common in some isolated sites. We propose the hypothesis that this patchy distribution of *G*. *poeciloptera* can be explained by the fact that the natural exposure of the C-horizon is infrequent in the tropical region, where well developed, deep and permeable soils are common [35, 36]. Therefore, soil attributes must be considered when investigating the driving forces that shape the distribution and abundance of tropical birds that nest in soil cavities.

## Supporting information

S1 File Raw data. In this file we include all the data used in this paper, organized by the relationships tested.
(PDF)
