# Supplementary material for: Soil attributes drive nest-site selection by the campo miner Geositta poeciloptera
Source: PLoS One. 2018 Jan 30;13(1):e0192185. doi: 10.1371/journal.pone.0192185 (PMC5790285; doi:10.1371/journal.pone.0192185)
Supplement: S1 File — In this file we include all the data used in this paper, organized by the relationships tested. (PDF) [file pone.0192185.s001.pdf]

Supporting information for Meireles et al. 2018. Soil attributes drive nest-site selection by the Campo Miner *Geositta poeciloptera*. PLoS One.

Here we include all the data used in this paper, organized by the relationships tested.

1) Relationship between nest depth and soil resistance

Nest	Nest depth (cm)	Soil resistence (MPa)
1	42	0.19
2	42	0.07
3	49	0.11
4	58	0.04
5	40	0.04
6	61	0.12
7	40	0.25
8	42	0.10
9	39	0.25
10	30	0.07
11	39	0.19
12	48	0.12
13	40	0.14
14	42	0.15
15	33	0.30
16	39	0.18
17	44	0.09
18	38	0.20
19	59	0.09
20	52	0.12
21	37	0.27
22	34	0.25
23	41	0.07
24	40	0.33
25	40	0.22
26	41	0.25
27	39	0.19
28	42	0.21
29	31	0.27
30	64	0.10
31	41	0.13
32	39	0.18
33	36	0.34
34	48	0.12
35	42	0.07
36	44	0.11
37	35	0.13
38	39	0.25

39	44	0.20
40	37	0.08
41	51	0.21
42	36	0.26
43	34	0.21

2) Percentage of sand, silt and clay found on the soil where each nest was constructed

Nest	% Sand	% Silt	% Clay
1	0.181	0.59	0.229
2	0.263	0.663	0.074
3	0.476	0.482	0.042
4	0.338	0.534	0.128
5	0.52	0.381	0.098
6	0.289	0.552	0.159
7	0.547	0.414	0.04
8	0.464	0.235	0.301
9	0.127	0.678	0.195
10	0.172	0.74	0.088
11	0.438	0.512	0.05
12	0.276	0.182	0.541
13	0.258	0.554	0.188
14	0.22	0.707	0.073
15	0.517	0.314	0.17
16	0.105	0.785	0.11
17	0.202	0.716	0.082
18	0.226	0.559	0.215
19	0.511	0.427	0.062
20	0.177	0.575	0.249
21	0.376	0.556	0.068
22	0.13	0.696	0.174
23	0.13	0.643	0.228
24	0.186	0.234	0.58
25	0.155	0.293	0.552
26	0.248	0.667	0.085
27	0.438	0.512	0.05
28	0.27	0.541	0.188
29	0.331	0.393	0.275
30	0.632	0.313	0.056
31	0.269	0.532	0.199
32	0.105	0.785	0.11
33	0.313	0.591	0.095
34	0.276	0.182	0.541
35	0.564	0.257	0.179
36	0.234	0.55	0.216

37	0.269	0.598	0.132
38	0.287	0.135	0.579
39	0.606	0.26	0.134
40	0.185	0.475	0.34
41	0.653	0.273	0.074
42	0.171	0.3	0.529
43	0.276	0.497	0.227

3) Horizon tickness of each bank with and without nest and also of each of the forty-three nests.

Banks without nest	B Horizon (cm)	C Horizon (cm)*	Total Height
1	39	44	83
2	45	16	61
3	4	101	105
4	81	0	81
5	28	33	61
6	42	106	148
7	56	201	257
8	58	51	109
9	48	29	77
10	11	47	58
11	34	26	60
12	8	71	79
13	8	105	113
14	57	33	90
15	32	32	64
16	71	10	81
17	31	25	56
18	57	62	119
19	48	14	62
20	32	43	75
21	25	36	61
22	52	14	66
23	11	45	56
24	15	66	81
25	39	46	85
**Banks with nest**	**B Horizon (cm)**	**C Horizon (cm) ***	**Total Height**
1	27	67	94
2	59	77	136
3	32	61	93
4	88	0	88
5	29	51	80
6	26	36	62
7	2	114	116
8	3	96	99
9	3	279	282

10	142	0	142
11	18	68	86
12	0	91	91
13	24	46	70
14	59	19	78
15	10	86	96
16	31	78	109
17	30	141	171
18	11	90	101
19	0	75	75
20	23	101	124
21	3	60	63
22	18	95	113
23	38	83	121
24	44	276	320
25	5	130	135

Nest	B Horizon (cm)	C Horizon (cm) *	Total Height
1	8	45.5	53.5
2	21	133	154
3	34	121.5	155.5
4	15	103.5	118.5
5	28	109	137
6	91	0	91
7	5	47	52
8	41	115.5	156.5
9	8	94	102
10	8	113	121
11	38	119	157
12	32	127	159
13	93	0	93
14	24	65	89
15	34	93	127
16	37	77	114
17	29	109	138
18	3	58	61
19	19	83	102
20	0	105	105
21	32	121	153
22	54	413	467
23	21	101	122
24	15	164	179
25	23	59	82
26	22	41	63
27	24	74	98
28	32	127	159
29	14	188	202
30	0	146.5	146.5
31	34	244	278

32	3	58	61
33	29	109	138
34	3	76	79
35	24	164	188
36	18	123	141
37	31	52	83
38	41	62	103
39	167	66	233
40	32	103	135
41	0	86	86
42	27	52.5	79.5
43	13	196	209

*Include BC and Cr Horizon.
